# Gene Expression Comparison between Sézary Syndrome and Lymphocytic-Variant Hypereosinophilic Syndrome Refines Biomarkers for Sézary Syndrome

**DOI:** 10.3390/cells9091992

**Published:** 2020-08-29

**Authors:** Andrea Moerman-Herzog, Syed J. Mehdi, Henry K. Wong

**Affiliations:** Department of Dermatology, University of Arkansas for Medical Sciences, 4301 W. Markham Street, Little Rock, AR 72205, USA; MoermanAndreaM@uams.edu (A.M.-H.); SJMehdi@uams.edu (S.J.M.)

**Keywords:** Sézary syndrome, cutaneous T-cell lymphoma, lymphocytic-variant hypereosinophilic syndrome, L-HES, transcriptome, biomarker, disease control

## Abstract

Sézary syndrome (SS), an aggressive cutaneous T-cell lymphoma (CTCL) with poor prognosis, is characterized by the clinical hallmarks of circulating malignant T cells, erythroderma and lymphadenopathy. However, highly variable clinical skin manifestations and similarities with benign mimickers can lead to significant diagnostic delay and inappropriate therapy that can lead to disease progression and mortality. SS has been the focus of numerous transcriptomic-profiling studies to identify sensitive and specific diagnostic and prognostic biomarkers. Benign inflammatory disease controls (e.g., psoriasis, atopic dermatitis) have served to identify chronic inflammatory phenotypes in gene expression profiles, but provide limited insight into the lymphoproliferative and oncogenic roles of abnormal gene expression in SS. This perspective was recently clarified by a transcriptome meta-analysis comparing SS and lymphocytic-variant hypereosinophilic syndrome, a benign yet often clonal T-cell lymphoproliferation, with clinical features similar to SS. Here we review the rationale for selecting lymphocytic-variant hypereosinophilic syndrome (L-HES) as a disease control for SS, and discuss differentially expressed genes that may distinguish benign from malignant lymphoproliferative phenotypes, including additional context from prior gene expression studies to improve understanding of genes important in SS.

## 1. Introduction

Cutaneous T-cell lymphomas (CTCL) represent a heterogeneous group of skin homing T-cell malignancies, with mycosis fungoides (MF) and its leukemic variant Sézary syndrome (SS) accounting for the majority of cases. These two variants present with highly variable clinical skin inflammation that can be mistaken for benign mimickers, such as psoriasis, atopic dermatitis and other benign inflammatory dermatoses (BID) [1,2,3]. A correct diagnosis of MF/SS can be delayed for many years, potentially leading to inappropriate therapy, disease progression, and death [4,5]. MF and SS are classified by the clinical staging system jointly established by the World Health Organization and the European Organization for Research and Treatment of Cancer (WHO-EORTC) [6,7]. Diagnosis can be challenging, and currently relies on a combination of several nonspecific clinical, histopathologic and diagnostic criteria [2,5,6,8]. Presently, there remains a need to identify reliable diagnostic, stage-associated and prognostic biomarkers of MF/SS to improve initial diagnosis and identify patients at risk for progression.

Gene expression biomarkers have enormous potential to improve clinical practice for MF/SS, and innovative efforts have been taken to identify sensitive and specific diagnostic and prognostic biomarkers [9,10,11,12]. Studies have employed “disease controls” like psoriasis and atopic dermatitis to filter out gene expression signals contributed by inflammatory processes [11,13,14,15,16,17]. Many BIDs display some clinical, histologic and immunologic features that resemble MF/SS [1,2,18]. While MF and SS are caused by neoplastic T cells, benign/reactive T cells play a prominent role in inflammatory symptoms and contribute inflammatory gene expression signatures that may confound early diagnosis of MF/SS [19]. The inflammatory milieu of late MF and SS acquires a T helper type-2 (Th2)-biased phenotype, with additional regulatory and Th17-like features [20]. SS shares many similarities with atopic dermatitis, which is also characterized by Th2 skin inflammation, and can acquire additional Th-phenotypes in chronic disease (reviewed by Saulite et al. [18]). While Th2 skin inflammation is common, Th1 and Th17 infiltrates are typical of psoriasis, and the inflammatory phenotype of contact dermatitis varies with exposure [21] (Table 1). Nevertheless, comparing the T cell phenotypes of BIDs to the dysregulated T cell phenotypes in CTCL has contributed important information to gene expression studies. Boonk et al. [8] effectively demonstrated that cases of benign erythroderma express some SS-biomarker genes at levels that exceed healthy donors, while the most specific SS-biomarker genes showed the least overlap in mRNA expression between SS and benign erythroderma cases. Inclusion of disease controls for benign/reactive inflammation can therefore improve the specificity of gene expression data to identify highly relevant genes unique to MF and/or SS, and exclude genes that could misclassify patients. 

A remaining challenge to the understanding of SS is the lack of a well-defined, pre/early-neoplastic T-cell population suitable for studies of stage progression, and the evolution of neoplastic phenotypes. However, this role was recently filled by a benign T-cell lymphoproliferative disorder known as lymphocytic-variant hypereosinophilic syndrome (L-HES) [22], which has skin inflammation and hematologic abnormalities that resemble SS [23,24,25,26,27,28,29]. A dominant T-cell clone in the blood is detected in a large majority of L-HES cases [23,25], and atypical T cells often exceed 70% of circulating lymphocytes [30]. While clonal T-cell populations can occasionally be detected in BIDs, they are not typical, nor a defining feature [12]. BIDs are characterized by polyclonal infiltrates of reactive T cells, and are not considered cutaneous lymphoproliferative disorders [31,32]. The combination of benign inflammatory and clonal lymphoproliferative phenotypes makes L-HES a unique disease control (Table 1) that can provide meaningful biological context that was previously lacking in transcriptomic studies of SS [22]. 

In this review, we will present the clinical and molecular similarities between SS and L-HES, discuss the rationale for using L-HES as a disease control for SS, and review the genes highlighted in a recent meta-analysis of SS and L-HES transcriptomes. We will also incorporate into perspective prior gene expression and functional studies of SS that together may distinguish characteristics of malignant and benign lymphoproliferative phenotypes, and their significance.

## 2. Clinical Features of SS and L-HES

**Figure 1:**
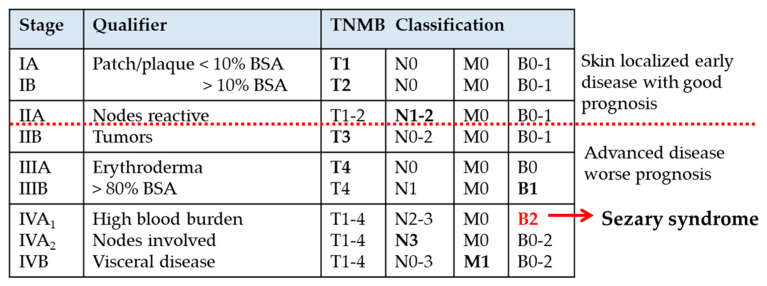
Clinical criteria for staging of mycosis fungoides (MF) and Sézary syndrome (SS). SS is classified as stage IVA_1_ disease and is distinguished from MF by a high blood tumor burden. Diagnostic “B2” blood criteria required for SS include Sézary cells ≥ 1000 cells/μL, CD4/CD8 ≥ 10, CD4^+^CD7^−^ cells > 30% or CD4^+^CD7^−^ cells ≥ 40%, with an identical T-cell clone detected in blood and skin. Stage IIIB requires B1 blood involvement not meeting the B2 threshold for SS. The most distinguishing qualifier for each stage is noted. The table is adapted from the ISCL/European Organization for Research and Treatment of Cancer (EORTC) classification criteria in Olsen et al. [33]. BSA, body surface area affected. Erythroderma, BSA > 80%.

Early MF presents with limited skin involvement and an indolent clinical course that may last for decades. Disease progression can include an increase in affected skin area, thickening of skin lesions from flat patches and plaques to nodular tumors, and the spread of malignant T cells to lymph nodes and visceral tissues (Figure 1) [33]. A subset of MF patients develops limited blood involvement (stage IIIB), and some may progress to frank SS satisfying B2 blood criteria (stage IVA_1_, Figure 1). SS is an advanced, leukemic variant of MF, distinguished from MF by generalized erythroderma and a high burden of tumor cells circulating in peripheral blood (Figure 1). However, the vast majority of SS cases arise de novo, without prior definable skin lesions characteristic of MF. This observation is consistent with the notion that MF and SS arise from distinct memory T cell subsets [34]. Most cases of SS share a classic triad of symptoms: pruritic erythroderma, lymphadenopathy, and a high burden of circulating clonal T cells. The B2 threshold for blood involvement in SS is met by Sézary cells in excess of 1000 cells/μL, CD4/CD8 ≥ 10, CD4^+^CD7^−^ cells ≥ 30%, or CD4^+^CD26^−^ cells ≥ 40% [6,33].

While SS is a moderately aggressive peripheral T-cell lymphoma with a 5-year overall survival rate of 36% [7], L-HES is typically indolent [25,35]. Chronic L-HES is infrequently associated with cytogenetic changes [23,25], whereas SS T cells harbor frequent chromosomal abnormalities and widespread changes in epigenetic status that can alter gene expression and prognosis (Table 2) [36,37,38,39,40,41]. Despite these important differences, SS and L-HES have similarities in a number of clinical and molecular findings (Table 2). In both SS and L-HES, eosinophils and abnormal T cells can be detected in both blood and skin, and over two thirds of L-HES patients experience skin inflammation and pruritus [25,27]. While a clonal T cell population is detected in the majority of L-HES cases, there is no consensus threshold for circulating, abnormal T cells, which show variable immunophenotypes, most often CD3^−^CD4^+^, with frequent CD7 loss similar to SS (Table 2) [24,35,42]. In contrast, CD26 loss is common to SS but has not been reported in L-HES. Importantly, prior studies have shown that L-HES CD3^−^CD4^+^ T cells have a CD45RO^+^ memory phenotype [29,30,42], which is shared by the majority of abnormal T cells in SS [43].

Causes of eosinophilia can be broadly classified as primary/neoplastic or secondary/reactive. Primary eosinophilias are defined by the presence of clonal, neoplastic eosinophils, such as in chronic eosinophilic leukemia or myeloproliferative HES [44]. Neoplastic eosinophil/myeloid clones may bear cytogenetic abnormalities such as the FIP1-like-1-platelet-derived growth factor receptor-alpha (*FIP1L1-PDGFRA*) fusion, which is effectively targeted by imatinib therapy [45]. Secondary eosinophilias are polyclonal and reactive, and are thought to depend on cytokines and growth factors that support eosinophil maturation and/or proliferation. They may be the result of parasitic or other infectious, allergic diseases, or lymphoid neoplasms and lymphoproliferations, which include L-HES [29]. L-HES is defined by early and severe eosinophilia secondary to an over proliferation of abnormal, and often clonal, Th2 T-cells secreting the eosinophilopoietic cytokine interleukin-5 (IL-5). An important diagnostic criterion for L-HES is eosinophilia exceeding 1500/µL in blood. Similarly, blood eosinophils are elevated in a subset of SS patients, and eosinophilia >700/μL is an indicator for disease progression in SS (Table 2) [46]. Increased eosinophils, particularly in the skin, have been associated with activation of signal transducer and activator of transcription 3 (STAT3) in CTCL T cells [47,48], and additional Janus kinase (JAK)-STAT dysfunction is frequent in CTCL, other T-cell leukemia/lymphomas, and many BIDs [49,50]. STAT3 is required for Th2 T-cell differentiation [51], and mediates IL-5 production in CTCL cell lines [52]. The discovery of an activating mutation of STAT3 in L-HES suggests a role for STAT3 dysfunction in the Th2 phenotype typical of L-HES [53]. These findings underscore the similarities in SS and L-HES at the molecular level. In addition, certain therapies are effective for both SS and L-HES, such as interferon (IFN)-α (Table 2). IFN-α has been shown to suppress IL-5 production in peripheral blood mononuclear cells (PBMCs) obtained from patients with eosinophilic SS [54]. Thus, while SS, L-HES, and BIDs are all T-cell disorders with clinical skin inflammation (Table 1), L-HES shares lymphoproliferative and eosinophilic features with SS that are absent in classical BIDs like atopic dermatitis (Table 2).

## 3. Gene Expression in SS and L-HES

SS gene expression profiles have been explored in a number of transcriptomic studies [14,16,17,68,71,72,73,74,75,76], and several multi-gene panels have been proposed to distinguish SS from BID with high accuracy [8,15,16,77], detect SS patients with as few as 5% circulating tumor cells [71], or offer prognostic insight [11]. However, these comparisons lacked the ability to exclude Th2 and lymphoproliferation genes, which may be expressed in both SS and L-HES, as L-HES is a Th2 lymphoproliferation.

There are two published transcriptomic studies of atypical T cells from L-HES patients. Ravoet et al. [30] observed abnormal expression of 850 genes in L-HES CD3^−^CD4^+^ T cells, with notable changes for growth control genes, including abnormally high expression of *IL17RB* (IL-25 receptor) and altered expression of transforming growth factor-β superfamily genes. Walker et al. [53] described significant upregulation of a STAT3-target gene signature, which may contribute to the Th2-like phenotype of L-HES T cells. 

**Figure 2:**
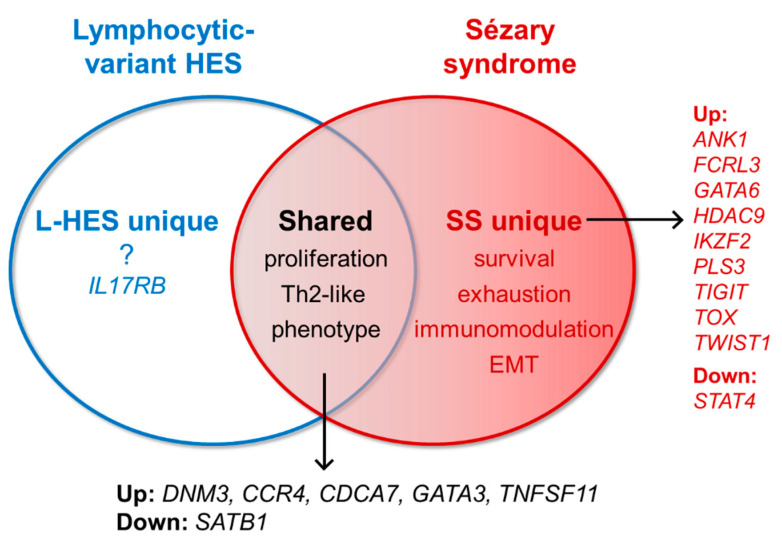
Overlap of differentially expressed genes for L-HES and SS. Shared and unique gene expression in SS and L-HES are shown with relationships to their respective roles in disease, which may lead to diagnostic improvements. Red circle: Differential gene expression in SS CD3^+^CD4^+^CD45RO^+^ T cells compared to normal CD3^+^CD4^+^CD45RO^+^ T cells [22]. Blue circle: Differential gene expression in L-HES CD3^−^CD4^+^ T cells compared to normal CD3^+^CD4^+^ T cells [30]. Center overlap: Gene expression abnormalities shared by SS and L-HES may reflect benign or pre-neoplastic proliferative and inflammatory phenotypes. Excluded areas to the right and left represent abnormal gene expression unique to SS or L-HES, respectively. SS-unique genes have been associated with a number of cancer-promoting phenotypes.

The public L-HES data set from Ravoet et al. [30] was recently compared to gene expression data from SS memory T cells [22] (Figure 2). Importantly, both data sets were obtained on the same microarray platform. The outcome of this meta-analysis approach was greater confidence in the identification of biomarker genes specific to the malignant phenotype of SS T cells, which eliminated Th2- and lymphoproliferation-associated genes inherent to L-HES. A common analysis workflow was used for both data sets to identify genes of interest, and changes in SS or L-HES gene expression compared to normal donors was based on a threshold of 2-fold with q ≤ 0.05 [22]. The outcome showed a highly significant degree of overlap between the abnormal gene expression profiles of SS and L-HES T cells compared to normal T cells (Figure 2), suggesting that gene expression shared by SS and L-HES reflects benign lymphoproliferative and Th2 phenotypes rather than malignant processes. Interestingly, shared genes included *DNM3*, *CCR4* and *GATA3*, which have appeared in diagnostic and prognostic gene-expression panels previously proposed for SS [8,11,15,16]. Nevertheless, L-HES and SS are distinct diseases, and gene expression abnormalities unique to either SS or L-HES were also identified by the meta-analysis (Figure 2). Many frequently published SS-biomarker genes were found in the “SS-unique” group, including increased expression of *PLS3*, *TOX* and *TWIST1*, and reduced expression of *STAT4*, confirming their association with malignancy. By identifying groups of shared and SS-unique genes associated with benign and malignant phenotypes, respectively, this novel comparison offered a new perspective on abnormal gene expression in SS. 

A limitation of the meta-analysis is the small number of cases included for both SS and L-HES. However, the SS and L-HES subjects have well-annotated clinical and immune characteristics that increase confidence in the results. In addition, we conducted a literature review of ten prior SS transcriptome studies (Table 3) to determine to what extent the SS-unique genes have been identified in other SS cohorts. Of the studies used for comparison, seven used microarrays, three used bulk RNA sequencing, and one used single-cell RNA sequencing. Eight studies compared SS patients to healthy donors, and one study included BIDs. Two studies compared malignant SS cells to non-malignant cells from the same patients. 

**Figure 3:**
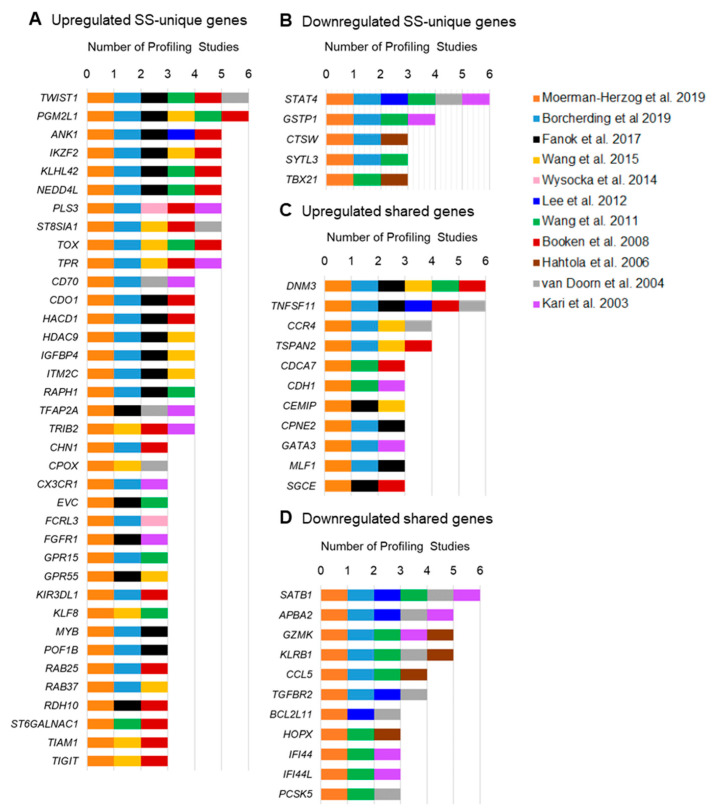
Differentially identified genes from the meta-analysis of SS and L-HES are supported by prior SS studies. Gene expression results from Moerman-Herzog et al. were compared to prior transcriptomic profiling studies of SS (Table 3). Genes differentially expressed from SS of prior studies were identified from the manuscript and supplementary data, using the significance threshold defined by each study. Gene symbols were updated using the Molecular Signatures database [79] and/or the GeneCards database [80]. Gene groups are defined by expression pattern, (**A**) upregulated SS-unique genes, (**B**) downregulated SS-unique genes, (**C**) upregulated shared genes, (**D**) downregulated shared genes. Only genes reported in at least three studies are shown. For each gene, studies that reported significant differential expression for that gene are represented by color-coded boxes next to the gene symbol.

After comparing results of the meta-analysis to the ten other studies in Table 3, we determined that thirty-seven upregulated and five downregulated SS-unique genes have also been reported in at least two additional transcriptomic studies of SS (Figure 3). The ten SS-unique genes most frequently reported as upregulated in other SS cohorts are *TWIST1*, *PGN2L1*, *ANK1*, *IKZF2*, *KLHL42*, *NEDD4L*, *PLS3*, *ST8SIA1*, *TOX*, and *TPR* (Figure 3A). Each of these genes has been reported in at least four other publications. SS-unique genes frequently reported as downregulated in other SS cohorts include *STAT4*, *GSTP1*, *CTSW*, *SYTL3*, and *TBX21* (Figure 3B). The small number of downregulated SS-unique genes supported by multiple other studies may reflect under-reporting of downregulated genes in the literature, as no supplemental data were available for downregulated genes from three studies [16,68,73]. 

We also compared genes abnormally expressed in L-HES [22,30] with other SS studies from Table 3 to identify gene expression shared by multiple SS cohorts. For genes identified as shared between SS and L-HES by the meta-analysis, eleven upregulated and eleven downregulated genes were reported in at least two other transcriptomic studies of SS (Figure 3C,D). Upregulated shared genes include *DNM3*, *TNFSF11*, *CCR4*, *TSPAN*, *CDCA7*, *CDH1*, *CMIP*, *CPNE2*, *GATA3*, *MLF1*, and *SGCE* (Figure 3C), and downregulated shared genes include *SATB1*, *APBA2*, *GZMK*, *KLRB1*, *CCL5*, *TGFBR2*, *BCL2L11*, *HOPX*, *IFI44*, and *PCSK5* (Figure 3D). We also identified seven genes upregulated in L-HES that were not shared with the SS cohort from the meta-analysis, but were concordantly differentially expressed with at least two prior transcriptomic studies for SS. These genes include *CCDC167* [68,74], *DUSP4* [16,73], *LMNA* [73,74], *NINJ2* [71,74], *PTTG1* [17,74], *TNFSF10* [68,71], and *GPR171* [16,17]. Thus, many of the shared and SS-unique genes identified by the meta-analysis of SS and L-HES gene expression are supported by prior studies in SS. How well the L-HES transcriptome data of Ravoet et al. represent other L-HES cohorts will remain an open question until additional studies are performed or added to public data repositories. The remainder of this review will consider the potential functional roles of shared and unique gene expression in SS. 

### 3.1. Gene Expression Shared by SS and L-HES

While genes with expression changes common to SS and L-HES are not ideal diagnostic biomarkers, they can provide additional insight into molecular mechanisms that support similarities in disease phenotype, and may have prognostic value. Increased expression of *GATA3* and decreased expression of *SATB1* are important examples. GATA3 is a Zn-finger transcription factor and master regulator of Th2 differentiation, and both *GATA3* and Th2 cytokine genes are frequently overexpressed in both SS and L-HES [30,53,71,74,81,82]. The Th2 cytokine IL-5 promotes eosinophilia in SS and L-HES, and activation of the *IL5* promoter by GATA3 is directly opposed by the transcription factor SATB1 [83]. Decreased expression of *SATB1* observed in both SS and L-HES reduces this check on *IL5* expression. 

Skin homing is another phenotype common to both SS and L-HES T cells, which is mediated by CCR4, a chemokine receptor essential for cutaneous homing of T cells [84]. Skin-resident SS T cells have a higher proliferative index than circulating SS cells, which may be dependent on cytokines and other factors present in the skin microenvironment [85]. The active role of CCR4 in SS is shown by the efficacy of anti-CCR4 immunotherapy approved for use in CTCL [86]. CCR4 expression is also suppressed in SS T cells and/or cell lines by other therapies with efficacy in CTCL, including the rexinoid bexarotene and histone deacetylase inhibitors (HDACi) romidepsin and suberoylanilide hydroxamic acid (SAHA/vorinostat) [87,88].

Genes with potential roles in lymphoproliferation were also concordantly regulated in SS and L-HES, including increased expression of *CDCA7*, *DNM3* and *TNFSF11*, and decreased expression of *SATB1*. SATB1 can sensitize SS cells to activation induced cell death [72], and low expression of *SATB1* is an independent prognostic factor in CTCL [89]. *TNFSF11* encodes receptor activator nuclear factor-κB ligand (RANKL), which is frequently expressed by cancer cells. The many roles for RANKL-RANK signaling in cancer-niche development, metastatic processes, neoangiogenesis, and immune escape have been reviewed recently [90]. The shared expression of *TNFSF11* in SS and L-HES is consistent with the enhanced proliferation of T cells. Increased expression of *DNM3* was associated with a better overall survival in a large cohort of SS patients [91], which is supported by its ability to inhibit colony formation and increase the expression of p53 protein in hepatocellular carcinoma cell lines [92]. The shared expression of *DNM3* in SS and L-HES is surprising because *DNM3* is a component of two multi-gene panels that differentiated SS from MF and BID cases including psoriasis, atopic dermatitis, and benign erythroderma with sensitivity and specificity over 95% [8,16].

*CDCA7* is a shared gene with a previously unrecognized role in SS, despite its detection in several prior transcriptomic studies of SS [16,72,74]. *CDCA7* is frequently overexpressed in human cancers [93], including a large variety of B- and T-cell leukemias and lymphomas, and its loss has been shown to reduce T- and B-lymphomagenesis in vivo [94,95]. Thus, *CDCA7* expression may be important to the proliferative nature of SS and L-HES. CDCA7 has weak transforming activity of its own, and is a direct target of C-Myc oncoprotein [96]. CDCA7 also contributes to anchorage-independent growth [94], and silencing *CDCA7* impaired lymphoma cell migration and invasion in in vitro and in vivo models [95]. A single cell RNA sequencing study of malignant and nonmalignant T cells from one SS patient revealed that *CDCA7* expression was an early event in the evolution of heterogeneous transcriptional states in SS clonal T cells [74], suggesting that high *CDCA7* expression may provide a receptive environment for additional changes. In summary, many gene expression abnormalities shared by SS and L-HES T cells appear to fulfill roles that promote Th2-like and proliferative phenotypes. While SS biomarkers *DNM3* and *SATB1* may have promise as prognostic biomarkers, they do not reflect processes unique to malignant T cells.

### 3.2. Gene Expression Unique to SS

The meta-analysis strategy comparing SS and L-HES is supported by the inclusion in the SS-unique category of many genes with rich publication histories in SS, such as *PLS3*, *TWIST1* and *STAT4*. In addition, the potential importance of some less well-recognized SS biomarker genes has been elevated by their new SS-unique status. SS-unique genes are associated with several cancer-promoting mechanisms including enhanced survival, oncogenic mi-RNAs, T-cell exhaustion and immunoregulation.

#### 3.2.1. Well-Established SS-Unique Biomarker Genes

*PLS3* and *TWIST1* are two frequently reported and highly expressed SS-biomarker genes with established roles in the epithelial to mesenchymal transition, and frequent association with disease progression in solid malignancies. *PLS3* is one of the earliest SS biomarker genes identified and is consistently upregulated in the majority of SS cases [15,97,98], including SS without erythroderma [99], but is absent in the blood of normal donors, BID, and MF patients without blood involvement [10,97,98,100]. Because *PLS3* expression is limited to the CD26 negative T-cell population [98], and malignant T-cell clones [97], *PLS3* expression correlates with blood disease burden in SS [10]. In solid cancers, *PLS3* expression is a marker of circulating tumor cells with a metastatic phenotype, and this is associated with a poor prognosis [101,102]. In SS, *PLS3* expression has been associated with enhanced migration and apoptosis resistance [97]. However, in contrast to solid tumors, *PLS3* expression in SS is associated with a better prognosis [91]. Single cell RNA sequencing of SS T cells revealed that *PLS3* was expressed in only one of two divergent evolutionary transcriptional states that developed in a malignant T-cell clone [74].

Numerous studies have reported overexpression of *TWIST1* in SS [8,14,16,22,72]. TWIST1 is a highly conserved developmental transcription factor with diverse pathological functions in solid and hematological tumors [103,104]. In CTCL, the frequency and intensity of lesional TWIST1 expression was shown to increase with clinical stage, and was highest in SS; all late stage lesions that stained positive for TWIST1 were also positive for C-Myc [105]. This suggests that increased expression of TWIST1 plays a role in CTCL progression, and is consistent with the recent discovery that TWIST1 is involved in skin cancer initiation, maintenance and progression in a dose dependent manner [106]. TWIST1 is also an important suppressor of chronic Th1 inflammation [107,108], and ectopic expression of *Twist1* reduced effector cytokine expression in both Th1- and Th2-polarized cells [108]. This feature of TWIST1 may contribute to poorly inducible cytokine gene expression observed in SS cells [22,109]. In addition, the mechanism of *TWIST1* overexpression may vary between patients, resulting from promoter hypomethylation [110], or gain of chromosomal region 7p21.1 harboring the *TWIST1* gene [111].

Reduced *STAT4* expression is one of the most consistent findings in transcriptional studies of SS [8]. However, genes with reduced expression have limited usefulness as stand-alone biomarkers, and STAT4 has appeared in several multi-gene biomarker panels. Showe et al. [112] first described the loss of STAT4 expression in SS PBMCs, and Litvinov et al. [113] confirmed that low *STAT4* expression in CTCL lesional skin is associated with progressive disease. However, in a separate study, higher *STAT4* expression was associated with HDACi resistance in MF/SS patients [114], suggesting that alternative pathways to malignancy may be active in these patients. 

STAT4 expression loss in SS appears to be related to STAT5-mediated expression of oncogenic microRNA-155 (miR-155) [113,115], as knockdown of miR-155 in MF cell lines increased STAT4 expression and improved the apoptotic response to SAHA [113,116]. Ralfkiaer et al. [117] demonstrated that a microRNA panel including miRNA-155, miRNA-203 and miRNA-205 could accurately classify 95% of CTCL from BID cases. In addition to targeting *STAT4* mRNA, miR-155 increases mutations by simultaneously interfering with DNA repair and cell cycle checkpoints [118], and has been associated with several solid and hematological malignancies [119]. Phase 1 and phase 2 clinical trials are underway for Cobomarsen/MRG-106, a novel oligonucleotide inhibitor of miR-155 (NCT02580552, NCT03713320, NCT03837457), with promising interim results that emphasize the importance of miR-155 to CTCL pathogenesis [119]. 

In summary, *TWIST1*, *PLS3* and *STAT4* remain some of the most reliable diagnostic biomarkers of SS due to their selective dysregulation in malignant T cells, and absent expression in L-HES. Indeed, the combined altered expression of these three biomarker genes was demonstrated in a multicenter study to distinguish cases of SS from erythrodermic inflammatory dermatoses with 98% sensitivity and 100% specificity [8].

#### 3.2.2. SS-Unique Genes Associated with Regulatory and Exhaustion Phenotypes

It has been suggested that the phenotype of SS T cells is plastic and may respond to microenvironmental factors [43,120,121]. While it is well known that SS malignant T cells express Th2 cytokines, they also variably express characteristics of regulatory T cells (Tregs), including expression of forkhead box P3 (FoxP3), inhibitory receptors, and immunoregulatory cytokines [81,122]. Moreover, these FoxP3^+^ malignant T cells have suppressive activity [123], and are associated with a worse prognosis [124].

CTCL T cells from lesional skin also exhibit phenotypes of exhausted T cells, potentially due to persistent antigen stimulation in the skin microenvironment. Increased surface expression of compensatory inhibitory receptors has been detected in early stage skin lesions, and a positive correlation was found between increased gene expression for checkpoint receptors and higher disease stage [125]. The SS-unique genes *IKZF2*, *FCRL3*, *TIGIT* and *TOX* have each been associated with Tregs and/or T-cell exhaustion. FCRL3 is an orphan Fc-like receptor with immunoregulatory properties [126], and the inhibitory receptor TIGIT is expressed by chronically activated, exhausted T cells and activated Tregs [127,128]. Coexpression of TIGIT and FCRL3 identifies a population of highly suppressive, Helios^+^FoxP3^+^ conventional Tregs [129], and several studies have described elevated surface expression of TIGIT and FCRL3 on circulating CD4^+^ T cells in SS [78,122,130]. In addition, high expression of TIGIT or FCRL3 has been shown to correlate with CD26 loss and a high tumor burden [78,122], and clinical remission coincided with the disappearance of CD4^+^CD26^−^ T cells expressing FCRL3 [78], suggesting that the immunoregulatory phenotype of SS T cells plays an active role in disease.

The transcription factor TOX is required for the thymic development of all CD4 T-cell lineages [131], while in mature T cells, TOX is upregulated in exhausted T cells in the context of chronic antigen stimulation [132]. Strong TOX staining is observed in lesional skin from a large majority of SS and MF cases, but is much less common in BID skin. High *TOX* expression correlates with increased disease-specific mortality in SS [133], and predicts disease progression and poor survival in early MF [134]. Suppression of *TOX* expression increases cell cycle regulators and apoptosis in CTCL cell lines, and greatly reduces the growth of CTCL tumor xenografts in mice [133]. In addition, increased expression of both *TIGIT* and *TOX* was observed during the malignant progression of chronic L-HES to T-cell lymphoma [22]. These findings suggest that SS-unique gene expression is associated with regulatory and exhaustion phenotypes of malignant T cells in SS.

#### 3.2.3. New and Promising SS-Unique Biomarker Genes

Gene expression studies for SS have identified many differentially expressed genes, but there are additional SS-unique genes that merit further discussion. *ANK1*, *GATA6* and *HDAC9* have not been well characterized as SS biomarkers, but recent developments provide insight into their potential roles in SS. Overexpression of *ANK1* was detected by several gene expression profiling studies [16,75], and was independently validated by Moerman-Herzog et al. [22]. Importantly, the last intron of *ANK1* harbors *MIR486*, which is also overexpressed in SS, and is involved in cell survival [135]. Co-transcription of *ANK1* mRNA and miR-486 is activated in hematopoietic progenitor cells by MYB oncoprotein, in erythroid cells by GATA1 transcription factor, and in muscle cells by myocardin related transcription factor A (reviewed in [136]). In the meta-analysis of SS and L-HES [22], *MYB* was significantly upregulated in SS and down-regulated in L-HES, and the miRNA-486 target *MAF* [137] was significantly downregulated in SS and unchanged in L-HES. *ANK1* and miR-486 can also be upregulated in a p53 dependent manner following DNA damage. In this context, miRNA-486 promoted G2/M arrest, and ANK1 enhanced cell motility [138]. In the same study, high *ANK1* expression correlated with reduced survival in several cancers including chronic lymphocytic leukemia, and increased survival was demonstrated in patients with a high positive correlation between TP53 and *ANK1* expression.

The transcription factor GATA6 is widely associated with tumorigenesis [139], including an oncogenic role in CTCL [140]. In SS T cells, *GATA6* is overexpressed and directly induces expression of CD137L, which promotes proliferation, survival and migration of SS T cells and CTCL cell lines [22,110,140]. Higher numbers of GATA6^+^, CD137L^+^ and CD137^+^ cells have been detected in all stages of MF/SS lesional skin compared to normal skin [140]. Furthermore, expression of *GATA6* mRNA in SS T cells and CTCL cell lines is enhanced by DNA hypomethylation and histone acetylation [110,140], suggesting that ectopic *GATA6* expression is activated by epigenetic dysregulation common to CTCL T cells.

HDACi therapy differentially modifies the distinct chromatin accessibility signatures observed in leukemic and host T cells from CTCL patients. The only HDAC gene differentially expressed between host and SS cells is *HDAC9*, and the *HDAC9* locus is strongly accessible only in leukemic T cells [40]. Several studies have shown that HDAC9 limits the proliferation and suppressive potential of Tregs (reviewed in [141]), but promotes B cell lymphomagenesis [142], suggesting that the function of HDAC9 is context dependent. Interestingly, in vitro acquired resistance to ricolinostat, a selective HDAC6 inhibitor, was associated with higher HDAC9 expression in a B-cell lymphoma cell line [143], and HDAC9 expression has been associated with drug resistance and poor prognosis in a variety of solid malignancies [144,145]. Thus, *HDAC9*, *ANK1*, and *GATA6* are biomarkers with SS-unique expression discovered by the comparison of SS and L-HES that may illuminate important roles in SS pathogenesis.

## 4. Concluding Remarks

Identifying highly specific biomarkers for CTCL remains a challenge, but selecting informative controls can improve the outcome. SS and L-HES are skin-tropic diseases that share many clinical and immunological features that are mirrored in their gene expression profiles, which exhibit prominent Th2-like and lymphoproliferative signatures, more so than other inflammatory skin diseases. It is now clear that several previously described SS biomarkers are shared by L-HES, indicating that a reevaluation of their functional significance in oncogenesis unique to SS is needed. The remaining biomarker genes unique to SS are now more focused on malignant gene expression phenotypes, including immunomodulation, EMT, cell survival, and co-expression of oncogenic miRNAs. The refocused SS-unique gene set includes genes that have received little prior attention in SS, presenting an opportunity to gain insight from these less-studied genes [146] which may have potentially important roles in SS. One of the earliest gene expression profiling studies of SS, conducted by Kari et al. [71], noted that while as few as eight genes with high predictive power could be used to accurately classify SS patients with low tumor burden, the best 85 genes could be removed before classification dipped below 100%, and the remaining group of 300 genes with lower predictive power was still highly accurate. This suggests that expanded gene panels could mitigate the confounding effects of small sample size and disease heterogeneity, which have significantly impeded efforts to identify diagnostic gene expression panels that are both sensitive and specific in multiple cohorts, and to illuminate the underlying mechanisms of SS pathogenesis.

In closing, comparative analysis of SS and L-HES gene expression identified subsets of genes that are unique to each disease, and will serve to improve diagnostic accuracy. A new focus on gene expression associated only with the malignant T-cell phenotype in SS may also illuminate potential therapeutic targets for these T-cell diseases. Understanding the roles of these genes in SS, and the processes by which these genes become dysregulated will yield insight into the mechanisms driving these rare and diagnostically challenging diseases.

## Figures and Tables

**Table 1:** Shared and distinct features of Sézary syndrome and disease controls.

Disease Type	Skin Inflammation	Lympho-Proliferation	Malignancy
Sézary syndrome	Th2	clonal	+
L-HES	Th2	frequently clonal	−
Atopic Dermatitis	Th2	reactive	−
Psoriasis	Th1, Th17	reactive	−
Contact Dermatitis	Th1, Th2 or Th17	reactive	−

L-HES, lymphocytic-variant hypereosinophilic syndrome; Th1, T-helper type 1; Th2, T-helper type 2; Th17, T-helper type 17.

**Table 2:** Clinical and molecular features of Sézary syndrome and lymphocytic-variant hypereosinophilic syndrome (HES).

**Clinical Features**	**Sézary Syndrome**	**Lymphocytic-Variant HES**
Classification	lymphoma, stage IV	benign lymphoproliferation
Diagnostic criteria	Sézary cells > 1000/µL (or, CD4/CD8 ratio ≥ 10, CD4^+^CD7^−^ cells ≥ 40%, CD4^+^CD26^−^ cells ≥ 30%), with identical T-cell clone in blood + skin [6,55]	Rule out other causes of HES. Blood eosinophilia >1500/µL, abnormal T cells with no standardized threshold, frequent T-cell clonality, T cells secrete IL-5 [25,35,56].
Clinical course	moderately aggressive	indolent
Skin and physical symptoms	widespread erythroderma, pruritus, lymphadenopathy	limited erythroderma, urticaria, pruritus [24,27]
Residence of T cells	blood, skin, lymph node	blood, skin, lymph node, soft tissue [25]
Skin pathology	epidermotropic Sézary cells with cerebriform nuclei, eosinophils in some patients	abundant eosinophils, and perivascular, dermal infiltrate of small-medium size T cells with irregular nucleus and scarce cytoplasm [25,35]
Eosinophilia	some patients, late, moderate complications uncommon [29] >700/µL poor prognostic indicator [46]	all patients, early, severe, can cause organ damage
First line therapy	Systemic immunomodulation: ECP combined with interferons or other systemic (bexarotene, romidepsin, low dose methotrexate) and/or skin-directed (topicals, TSEBT) therapy [57,58]	systemic corticosteroids [35,59,60]
Second line therapy	Targeted and immune enhancing/sparing therapies preferred: mogamulizumab. romidepsin, alemtuzumab, intermediate dose methotrexate. Refractory disease: clinical trials, allogeneic HCT, chemotherapy [57,58]	IFN-α + glucocorticoids Steroid-sparing: mepolizumab, alemtuzumab, mycophenolate mofetil, cyclosporin, methotrexate, JAK kinase inhibitors (ruxolitinib, tofacitinib) [35,59,60] and imatinib, despite lack of FIP1L1-PDFGRA fusion, for patients who fail other tharapies [61]
Follow up	Monitor complete blood count with differential, liver function, LDH, flow cytometry for Sezary cells [33] in blood, physical examination for nodes, viscera and skin infections [62].	monitor T-cell lymphoma risk with lymphocyte counts, PB flow cytometry, BM cytogenetics [59]
Progression symptoms	Increases in pruritus, erythroderma, or skin tumor burden, enlarging lymph nodes, visceral organ involvement, immune suppression [63]	10–25% progress to T-cell lymphoma, cytogenetic changes *Nonspecific symptoms:* rapid increase in lymphocytosis, lymph node involvement, infiltrative nodules [25]
**Molecular Features**	**Sézary Syndrome**	**Lymphocytic-Variant** **HES**
T-cell phenotype	memory T cell with heterogeneous molecular phenotype [43,64]	memory T cell [30,42]
T-cell surface antigens	CD3^+/−^CD4^+^, CD7 and/or CD26 loss CLA^+^, CCR7^+^, CCR4^+^, CCR10^+^ [65,66]	CD3^−^CD4^+^CD7^−^CD5^++^, CD3^+^CD4^+^CD7^−^, or, CD3^+^CD4^−^CD8^−^ [23,35]
Cytokines	Th2 (IL-4, IL-5, IL-13), suppressive (IL-10), autocrine or paracrine growth stimulation (IL-15, IL-16, IL-32) [67,68]	Th2 (IL-4, IL-5, IL-13) [28,42]
Molecular drivers	Mutations in pathways related to DNA damage repair (*TP53*), apoptosis, (*FAS*), cell cycle (*MYC*, *RB1*), epigenetic modulators (*DNMT3A, TET2*), JAK/STAT (*JAK3, STAT3, STAT5B*), *ARID1A*, NF-κB (*NFKB2, CARD11*), TCR-signaling (*CD28, PLCG1*) [37,38,69,70]	IL-5, GATA3, JAK/STAT, IL17RB, TGFβ signaling [30,53]
Genetic abnormalities	Frequent SNV and CNV, C > T transitions consistent with UV damage, recurrent 10q and 17p deletions, recurrent 8q and 17q amplifications [37,38]; gene fusions [68]	Seldom reported, partial 6q deletion and other karyotype abnormalities [23]

BM, bone marrow; CNV, copy number variation; ECP, extracorporeal photopheresis; HCT, hematopoietic stem cell transplant; LDH, lactate dehydrogenase; ND, normal donor; PB, peripheral blood; PUVA, psoralen plus ultraviolet A; SNV, single nucleotide variation; TCR, T-cell receptor; TSEBT, total skin electron beam therapy.

**Table 3:** SS transcriptomic profiling studies included in Figure 3.

**Meta-Analysis Study**	**Sézary Patients**	**Healthy Donors**	**BID**	**Technology**
Moerman-Herzog et al. [22]	n = 3 CD3^+^CD4^+^CD45RO^+^	n = 3 CD3^+^CD4^+^CD45RO^+^	n.a.	microarray
**Prior Study**	**Sézary Patients**	**Healthy Donors**	**BID**	**Technology**
Fanok et al. [73]	n = 8 CD3^+^CD4^+^CD7^−^ and/or CD3^+^CD4^+^CD26^−^	n =4 CD3^+^CD4^+^CD45RO^+^	n.a.	RNAseq
Wang et al. [68]	n = 22 CD3^+^CD4^+^	n = 5 CD3^+^CD4^+^	n.a.	RNAseq
Wysocka et al. [78]	n = 6 CD3^+^CD4^+^	n = 3 CD3^+^CD4^+^	n.a.	microarray
Wang et al. [72]	n = 6 CD3^+^CD4^+^CD7-	n = 9 CD3^+^CD4^+^	n.a.	microarray
Booken et al. [16]	n = 10 PBMC	n =10 PBMC	n.a.	microarray
Hahtola et al. [17]	n = 4 PBMC	n = 5 PBMC	n.a.	microarray
van Doorn et al. [14]	n = 10 CD3^+^CD4^+^	n = 3 CD3^+^CD4^+^	n = 5 CD3^+^CD4^+^	microarray
Kari et al. [71]	n = 18 >60% CD4^+^	n = 12 Th2-skewed PBMC	n.a.	microarray
**Prior Study**	**Sézary Malignant Cells**	**Patient-Matched** **Non-Malignant Cells**	**BID**	**Technology**
Borcherding et al. [74]	n = 1 CD3^+^CD4^+^CD5^bright^SSC^hi^	n = 1 CD3^+^CD4^+^CD5^int^SSC^int^	n.a.	scRNAseq
Lee et al. [75]	n = 3 CD3^+^CD4^+^Vβ^+^	n = 3 CD3^+^CD4^+^Vβ^−^	n.a.	RNAseq

n.a., not applicable; RNAseq, RNA sequencing; scRNAseq, single cell RNAseq.
